# Targeting sphingolipid metabolism as an approach for combination therapies in haematological malignancies

**DOI:** 10.1038/s41420-018-0075-0

**Published:** 2018-06-28

**Authors:** Alexander C. Lewis, Craig T. Wallington-Beddoe, Jason A. Powell, Stuart M. Pitson

**Affiliations:** 1 Centre for Cancer Biology, University of South Australia and SA Pathology, UniSA CRI Building, North Terrace, Adelaide, SA 5001 Australia; 2 Adelaide Medical School, University of Adelaide, Adelaide, SA 5000 Australia; 3 Flinders Medical Centre, Bedford Park, SA 5042 Australia; 4 College of Medicine and Public Health, Flinders University, Bedford Park, SA 5042 Australia

**Keywords:** Leukaemia, Targeted therapies

## Abstract

Conventional chemotherapy-based drug combinations have, until recently, been the backbone of most therapeutic strategies for cancer. In a time of emerging rationale drug development, targeted therapies are beginning to be added to traditional chemotherapeutics to synergistically enhance clinical responses. Of note, the importance of pro-apoptotic ceramide in mediating the anti-cancer effects of these therapies is becoming more apparent. Furthermore, reduced cellular ceramide in favour of pro-survival sphingolipids correlates with tumorigenesis and most importantly, drug resistance. Thus, agents that manipulate sphingolipid metabolism have been explored as potential anti-cancer agents and have recently demonstrated exciting potential to augment the efficacy of anti-cancer therapeutics. This review examines the biology underpinning these observations and the potential use of sphingolipid manipulating agents in the context of existing and emerging therapies for haematological malignancies.

## Facts

• Efficacy of many chemotherapeutics and targeted therapies is dictated by cellular ceramide levels.

• Oncogene activation skews sphingolipid metabolism to favour the production of pro-survival sphingolipids.

• Inhibitors of enzymes involved in ceramide metabolism exhibit promise in the relapsed-refractory setting.

• Anti-cancer activity of sphingosine kinase inhibitors provides several options for new drug combinations.

**Open Questions**

• What other clinically utilised drugs rely on increases in ceramide levels for their efficacy and can they be effectively partnered with other ceramide inducing agents?

• How does ceramide modulate the Bcl-2 family proteins, Mcl-1 and Bcl-2?

• Are sphingolipid enzyme inhibitors best suited in the frontline or relapsed-refractory setting?

## Introduction

**Fig. 1:**
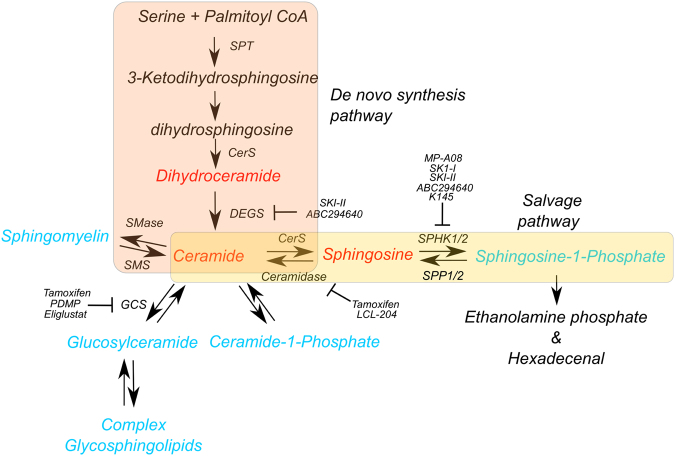
Overview of the sphingolipid cycle. The pleotropic nature of ceramide allows a promotion of multiple cellular fates including survival, migration and angiogenesis. Furthermore this also prevents a lethal accumulation of apoptotic sphingolipids (red) such as ceramide by maintaining a balance of pro-survival lipids (blue). Due to the propensity of transformed cells to deplete ceramide by increasing expression of enzymes, such as SPHK1/2 and GCS, inhibitors targeting these enzymes have exhibited therapeutic potential by tipping the balance to favour ceramide accumulation and promote cell death.

Frontline chemotherapeutic regimens for the majority of haematological malignancies have, until recently, undergone little change over the last 30 years. The success of tyrosine-kinase inhibitors (TKI) in chronic myeloid leukaemia (CML) has significantly increased the 10-year survival rate to 83%, enabling some patients to cease therapy (~40%) and achieve long-term remission (~40% >3 years)^1^. However, unlike CML which is driven solely by the BCR-ABL oncogene, most blood cancers are more genetically heterogeneous displaying complex clonal architecture with conventional chemotherapeutic agents remaining the backbone of most therapy regimens. One mechanism whereby chemotherapy induces apoptosis in malignant cells is through increases in the cellular levels of the pro-apoptotic sphingolipid, ceramide^2,3^. Sphingolipids are a class of lipids that can exert pleotropic cell signalling effects. Ceramide is a central component of sphingolipid metabolism that is tightly regulated due to its pro-apoptotic effects (Fig. 1). Enzymes involved in the conversion of ceramide to other sphingolipids have been implicated in drug resistance by depleting ceramide to produce pro-survival sphingolipids (Table 1). Indeed, inhibitors targeting these enzymes have been shown to induce cell death through inducing the accumulation of lethal levels of ceramide^4–7^. Thus, manipulating sphingolipid metabolism has shown promise in combination with conventional chemotherapy, as well as with novel agents. In this review, we highlight the literature that examines how targeting sphingolipid metabolism shows considerable promise for chemo-sensitising patients with blood cancers.

**Table 1:** Role of sphingolipid enzymes in haematological malignancies

**Enzyme**	**Malignancy**	**Role**
Acid ceramidase	AML	Increased expression in patient samples. Modulates Mcl-1 expression in a post-translational manner^4^
Ceramide synthase	AML	Suppressed by FLT3 signalling. Mediates cytotoxicity of FLT3 inhibitors by induction of lethal mitophagy^39^
Glucosylceramide synthase	AML	Overexpressed in chemotherapy resistance cell lines^29,30^
	CLL	Upregulated in response to B-cell receptor stimulation^68^
	Lymphoma	Potential role in tumour initiation^98^
	Myeloma	Potential role in tumour initiation^98^
Sphingosine kinase 1	AML	Overexpressed in patient samples. Increases drug resistance to chemotherapy and ceramide inducing strategies^16,18^
	ALL	Overexpressed in patient samples^78^
	CML	Overexpressed in Imatinib-resistant cell lines^15^. Upregulates Mcl-1 in a BCR-ABL dependent manner^60^. Represses PP2A to promote BCR-ABL stability^51^
Sphingosine kinase 2	ALL	Promotes B-ALL disease progression. Inhibits histone deacetylases to promote *Myc* expression^22^
	Myeloma	Upregulated in cell lines and patient samples^5,23^

*AML* acute myeloid leukaemia, *B-ALL* B cell acute lymphoblastic leukaemia, *BCR-ABL* Breakpoint cluster region–Abelson murine leukaemia viral oncogene homolog 1, *CLL* chronic lymphocytic leukaemia, *FLT3* FMS-like tyrosine kinase 3, *PP2A* protein phosphatase 2A

## The sphingolipid cycle

While originally assumed to just play an integral role in the structure of the cell membrane, sphingolipids have been found to be prominent players in cell signalling, capable of exerting a myriad of cell responses. De novo sphingolipid production commences at the endoplasmic reticulum with the condensation of serine and palmitoyl-CoA by the rate-determining enzyme, serine palmitoyltransferase to 3-keto-dihydrosphingosine (Fig. 1)^8^. Following further modification to dihydrosphingosine, ceramide synthases convert dihydrosphingosine to dihydroceramide which are then desaturated to generate ceramides^8^. From here, these ceramides can be modified to various sphingolipid species that modulate membrane composition and signal transduction. For example, ceramides can be glycosylated by glucosylceramide synthase to glucosylceramides which can serve as an intermediary for other glycosphingolipids, phosphorylated by ceramide kinase, modified by the addition of phosphocholine by sphingomyelinase (SMase) to form sphingomyelins, or deacylated by ceramidases to form sphingosine and subsequently, through the action of sphingosine kinases (SPHKs), generate sphingosine-1-phosphate (S1P) (Fig. 1)^9^. Notably, considerable evidence implicates important roles for several of these sphingolipid metabolic enzymes in tumorigenesis and resistance to therapy in haematological malignancies (Table 1).

### Sphingosine kinases

The “sphingolipid rheostat” is a concept that describes cell fate as a balance between pro-apoptotic ceramide and pro-survival S1P^9,10^. As the SPHKs are critical in the only exit point for degradation of sphingolipids, via conversion of sphingosine to S1P, and then its degradation by S1P lyase, these enzymes represent one of the key players in maintaining ceramide levels (Fig. 1). Furthermore, S1P generation by SPHK also directly promotes cell survival as well as activating oncogenic signalling pathways by acting as both an intracellular second messenger, and as a ligand for a family of five S1P-selective G-protein-coupled receptors^11^.

Although very similar enzymes, some sequence and presumed structural diversity between the two human SPHKs, SPHK1 and SPHK2, is thought to drive partially different biological functions of these enzymes^11^. It is generally accepted that SPHK1 is associated with a pro-tumorigenic role. Under homeostatic conditions, basal SPHK1 activity is thought to maintain ceramide levels in the absence of stimuli to prevent inappropriate cell death^12^. Activation of SPHK1 can occur via the RAS pathway with extracellular signal-regulated kinases 1/2 (ERK1/2) phosphorylating SPHK1 at Serine 225^12^. Activated SPHK1 subsequently translocates to the plasma membrane, via an interaction with calcium and integrin binding protein 1 (CIB1), to convert sphingosine to S1P^12–14^. Due to the frequency with which hyperactivation of the RAS pathway occurs in cancer, constitutive phosphorylation and activation of SPHK1 in cancer is likely to be common, promoting drug resistance by not only depleting ceramide levels but also promoting pro-survival signalling^9,15,16^. Furthermore, drug resistant cell lines have been shown to also exhibit increased SPHK1 activity and attenuate increases in ceramide which would otherwise promote apoptosis^15–17^. Several studies have shown that targeting SPHK1 can resensitise cells to chemotherapy, by blocking the conversion of sphingosine to S1P and generating a bolus of pro-apoptotic ceramide^15, 16,18^.

Despite catalysing the same reaction, characterising SPHK2 has proved more elusive with conflicting literature on its role in tumorigenesis^19,20^. Nevertheless, targeting SPHK2 has shown promise in a number of malignancies, including breast cancer^21^, acute lymphoblastic leukaemia (ALL)^22^ and myeloma^5,23^.

### Ceramidase

Breakdown of ceramide to sphingosine is mediated by ceramidases, with several homologues described that function in acidic, neutral, or alkaline pH. The most characterised form, acid ceramidase (AC) is thought to localise to acidic compartments, such as lysosomes^24^. The degradation of ceramide by AC has highlighted a potential mechanism for drug resistance by degrading the bolus of ceramide induced by cancer treatments. Overexpression of AC has been reported in several solid tumours^25^ in addition to acute myeloid leukaemia (AML)^4^. Pre-clinical studies utilising AC inhibitors have shown efficacy in resensitising cells to chemotherapeutics^26,27^. Although no direct AC inhibitors are currently under investigation in clinical trials, interestingly, the clinically available oestrogen receptor antagonist, Tamoxifen has demonstrated AC inhibition, warranting potential investigation as an adjuvant for chemotherapy regimens^28^.

### Glucosylceramide synthase

Glucosylceramide synthesis is the first step in the generation of complex glycosphingolipids (Fig. 1). Much of the interest in glucosylceramide has focussed on its role as a “sink” for ceramide, with the action of glucosylceramide synthase (GCS) shunting ceramide through glucosylceramide and the glycosphingolipid pathway. It is no surprise that the removal of ceramide through this pathway has been proposed as a mechanism of drug resistance and multiple studies have identified GCS as a therapeutic target by preventing the accumulation of lethal levels of ceramide^29–32^. The approval of the GCS inhibitor, Eliglustat, for clinical use in the lysosomal storage disorder, Gaucher’s disease, suggests that GCS inhibition is well tolerated in humans^33^ and could provide a means to investigate combinational studies of GCS inhibition with chemotherapeutics and targeted agents.

### Ceramide synthase

In mammals, six different ceramide synthases (CerS1-6) have been recognized, each capable of generating varying ceramide species of differing fatty acyl chain lengths in both the de novo sphingolipid pathway as well as the conversion of sphingosine to ceramides in the so-called sphingolipid salvage pathway (Fig. 1)^34^. Several studies have demonstrated that CerS activity is crucial to the cancer cell killing efficacy of chemotherapeutics^35^, tumour necrosis factor-related apoptosis-inducing ligand (TRAIL)-induced apoptosis^36^, radiation^37^ and kinase inhibitors^38,39^. Although activation of CerS presents an attractive therapeutic target, CerS regulation remains poorly understood^40^.

## Cellular ceramides are increased by chemotherapy

Chemotherapy frequently promotes the accumulation of ceramide, which appears to contribute to induction of cancer cell death^41–43^. While the mechanisms responsible for this induction of ceramide depends on the chemotherapy employed, numerous studies have observed chemotherapy-induced activation of CerS and acid SMase, as well as inhibition of AC^41–44^. Notably, among the genes activated by p53 in response to chemotherapy, CerS5^37^, CerS6^45^ and neutral SMase^46^ are prominent. There is also evidence to suggest that the cysteine protease, Cathepsin B can degrade SK1 following p53 upregulation by genotoxic stress, increasing ceramide levels^47^. Thus, with the widespread effect of chemotherapy and p53 on manipulating sphingolipid metabolism, direct modulation of sphingolipid enzymes in combination with current agents may provide improved clinical outcomes.

## Mechanisms of ceramide-induced cell death

Ceramide exerts its tumour suppressive activities through multiple mechanisms, including activation of protein phosphatases 1 (PP1) and 2A (PP2A)^48^, and suppression of oncogenes such as Akt^49^, c-Myc^50^ and Bcr–Abl^51^. Perturbation of sphingolipid homeostasis and ceramide accumulation within cell membranes has also been reported to invoke pro-apoptotic signalling through activation of the unfolded protein response^52^, autophagy^52^ and mitophagy^39,53^. Furthermore, there is strong evidence suggesting that ceramide can directly initiate apoptosis by the formation of channels within the mitochondrial outer membrane, capable of facilitating the release of proteins, such as cytochrome c, apoptosis-inducing factor and second mitochondria-derived activator of caspases (SMAC)^54^.

## Targeting perturbations in sphingolipid metabolism in haematological malignancies

Clearly, sphingolipid metabolism is frequently dysregulated in haematological malignancies, and can confer resistance to many drugs currently employed to treat these diseases. Thus, there appears potential therapeutic benefit in combining sphingolipid modulators with clinical chemotherapeutics and novel therapies for the treatment of a range of blood cancers.

### Chronic myeloid leukaemia

The BCR-ABL inhibitor Imatinib and subsequent TKIs, Dasatinib and Nilotinib have dramatically improved survival rates for CML, with some patients even discontinuing treatment due to prolonged molecular remission status^55,56^. The emergence of ATP binding site mutations within BCR-ABL, such as the T315I mutant, however, confers resistance to most of these ATP-competitive inhibitors, and presents a key therapeutic issue^57^. The recent development of allosteric BCR-ABL inhibitors, GNF-2 and ABL001 may provide an alternative option in the future to prevent this^58^. Analysis of GNF-2 treated cells revealed an increase in ceramide levels suggesting BCR-ABL may suppress ceramide synthesis^59^. Augmentation of ceramide levels using the glucosylceramide synthase inhibitor D-*threo*-l-phenyl-2-decanoylamino-3-morpholino-1-propanol (PDMP), enhanced apoptosis and resensitised T315I mutant CML cells to both Imatinib and Nilotinib^59^ suggesting co-targeting BCR-ABL with sphingolipid modulating agents may be a novel strategy to further enhance the efficacy of TKI treatment.

Comparative analysis of Imatinib sensitive and resistant K562 CML cells revealed enhanced S1P generation and reduced ceramide levels as a consequence of SPHK1 upregulation compared to parental Imatinib sensitive K562 cells^15^, suggesting a potential role for SPHK1 in Imatinib resistance. Notably, overexpression of SPHK1 blocked the cytotoxic effects of Imatinib in sensitive K562 cells recapitulating the phenotype of Imatinib resistance^15^. Findings by Li et al. corroborated this relationship with BCR-ABL upregulating SPHK1 activity through MAPK, PI3K and JAK2 signalling suggesting a positive feedback loop^60^. Genetic and chemical targeting of SPHK1 also uncovered a positive relationship between SPHK1 and anti-apoptotic protein Mcl-1, whereby overexpression of SPHK1 increased Mcl-1 levels in a BCR-ABL dependent manner^60^.

The precise mechanism between SPHK1 and BCR-ABL stability was later uncovered with S1P receptor 2 (S1P_2_) supressing PP2A-mediated de-phosphorylation and subsequent degradation of BCR-ABL^51^. Targeting S1P/S1P_2_ signalling resensitised Imatinib-resistant K562 cells and T315I patient blasts to BCR-ABL inhibition^51^. Notably, elevated SPHK1 and S1P_2_ mRNA levels were found in T315I patient blasts used in this study which raises the question as to whether there is a correlation between these genes and BCR-ABL mutational status^51^.

BCR-ABL inhibitors, Dasatinib, Nilotinib and GFN-2 have also been shown to induce transcription of various CerS genes to upregulate ceramide production^59,61,62^. Intriguingly, Dasatinib treatment was associated with increases in CerS2,5,6 whereas Nilotinib was associated with increases in Cers5 expression^61,62^. Combining either Dasatinib or Nilotinib with the GCS inhibitor, PDMP, augmented apoptosis, presumably through synergistic increases in ceramide levels^61^. The interplay between BCR-ABL, SPHK1 and CerS highlights the oncogenic potential of BCR-ABL in skewing sphingolipid metabolism to favour a pro-survival phenotype, and highlights a potential therapeutic benefit for combining ceramide inducers with BCR-ABL inhibitors.

### Chronic lymphocytic leukaemia

Chronic lymphocytic leukaemia (CLL) is an indolent form of leukaemia with some patients not requiring treatment in their lifetime^63^. CLL patients with unfavourable genetic factors such as 17p deletion (del(17p)) however respond poorly to frontline therapy in part due to the absence of p53^64^. The addition of the Bcl-2 inhibitor, Venetoclax has been a paradigm shift for p53 null CLL patients with 79% of relapsed/refractory patients in a phase II clinical trial responding, 20% of whom exhibited undetectable disease by flow cytometry^65^. As always, resistance to drugs remains a problem with other Bcl-2 family members such as Mcl-1, identified as a marker of resistance to Venetoclax^66^. Although still effective in CLL patients, identifying other drugs to use alongside Venetoclax and prevent the emergence of resistance is crucial.

In the context of sphingolipid metabolism, the Bcl-2/Bcl-xl inhibitor Navitoclax was found to enhance CerS activity and increase C16 ceramide synthesis. Although, the mechanism remains to be confirmed, the authors to proposed that Bcl-2 inhibition allows Bak to interact with CerS5 or 6 resulting in the production of C16 ceramide^67^. Furthermore, combining Navitoclax with either the GCS inhibitor, PDMP, or the SPHK inhibitor, SKI-II-induced synergistic increases in ceramide and cell death^7^.

Interestingly, B-cell receptor (BCR) signalling in primary CLL cells has been shown to induce glucosylceramide generation, potentially blunting the efficacy of Rituximab treatment^68^. Schwamb et al. proposed that BCR signalling stimulates GCS transcription upon IgM treatment of primary CLL cells in a manner dependent on phosphoinositide 3-kinase (PI3K)δ and Bruton’s tyrosine kinase (BTK) activity^68^. Notably, treatment of CLL cells with the PI3Kδ inhibitor, Idelalisib or BTK inhibitor, Ibrutinib which are currently FDA approved for CLL^69^, abrogated increases in GCS transcription and synergised with the first generation Bcl-2/Bcl-xl inhibitor ABT-737^7,67,68^. These data suggest that combining ceramide-inducing strategies with Bcl-2 inhibitors may exhibit synergistic activity in CLL.

A recent study by Dielschneider et al. investigating the use of “lysosomal penetrating” or lysosomotropic agents in CLL uncovered an increase in S1P phosphatase 1 (SPP1) and sphingosine in primary CLL patient samples^70^ compared to normal B cells. Lysomotropic agents accumulate within lysosomes, promoting the release of lysosomal contents such as cathepsins and the initiation of non-apoptotic cell death^71^. As a natural detergent and lysomotropic agent, sphingosine has been shown to induce lysosome destabilisation^72^ and thus may explain the susceptibility of CLL cells to lysomotropic agents^70^. The addition of either sphingosine or SKI-II augmented the apoptotic response of the lysosomotropic agent Siramesine, against primary CLL cells^70^. The clinical relevance of the above findings by Dielschneider et al. are intriguing particularly with reports of the anti-CD20 antibody Obinutuzumab inducing lysosomal permeabilisation in B-cell malignancies, highlighting its potential use in combination with ceramide/sphingosine-generating agents^73^.

### Acute lymphoblastic leukaemia

There is disparity in treatment outcome for ALL with cure rates for adults at 30–40% despite paediatric cases being closer to 90%^74^. Poor risk subtypes such as Philadelphia (Ph)-like and Ph positive ALL are frequently observed in adults (~50%) and typically respond poorly to chemotherapy^75^. The addition of a TKI to standard chemotherapy for Ph positive patients has improved treatment response and survival rates for many of these patients with initial studies showing complete haematological response in ~90% of patients^76^. Like CML, relapsed Ph positive ALL patients exhibit similar mutations in BCR-ABL that impart TKI resistance, requiring a move to second and third generation BCR-ABL inhibitors.

A potential role for the SPHKs in ALL was first highlighted using studies with the pan SPHK inhibitor, SKI-II, in combination with the commonly used chemotherapeutic Vincristine^77^. SKI-II treatment induced cell death in ALL cell lines and primary lymphoblasts. As expected, inhibition of the SPHKs and the accompanying increase in ceramide synergised with Vincristine treatment^77^. However, combining the SPHK2 inhibitor, ABC294640 with Doxorubicin or Vincristine elicited only an additive effect suggesting the synergy observed may be a consequence of SPHK1 inhibition^22^.

A role for SPHK1 in contributing to the development of BCR-ABL driven ALL has been described. Gene expression data from two separate patient cohorts revealed a significant increase in SPHK1 expression in BCR-ABL positive ALL compared with BCR-ABL negative cases, highlighting a potential relationship that had previously been described in CML^17,60^. Deletion of SPHK1 in BCR-ABL positive, but not BCR-ABL negative murine ALL models delayed disease incidence implicating SPHK1 as a significant player in BCR-ABL driven ALL^78^. Furthermore, combining SPHK inhibitors, SK1-I, SKI-II or ABC294640 with Imatinib also induced synergistic cell death in BCR-ABL positive cell lines which collectively warrants further investigation^78^.

Enhanced SPHK2 protein levels and activity in ALL patient samples and cell lines when compared with normal B-cell progenitors has also been reported^22^. SPHK2 inhibition was associated with reductions in histone acetylation of the c-Myc promoter representing new evidence for an oncogenic role for SPHK2^22^. Reductions in c-Myc expression were observed in BCR-ABL transformed B-ALL cells from SPHK2 knockout mice, translating into increased overall survival in vivo^22^. These pre-clinical studies may provide the impetus to assess the addition of ABC294640 alongside BCR-ABL inhibitors in patients with Ph positive ALL.

### Acute myeloid leukaemia

Among drugs in the development pipeline for AML, selective FLT3 inhibitors Crenolanib and ASP2215 have exhibited impressive single-agent activity (ORR 52%, CR_C_ 41%) in a phase I/II study of relapsed/refractory AML^79^. Recently, work by Dany et al. examined the mechanism of action of FLT3 inhibitors, including Crenolanib, and observed reductions in C18-ceramide levels in FLT3 positive AML patient blasts^39^. The authors attributed this to striking reduction in CerS1 mRNA levels, suggesting an inverse relationship between FLT3 activity and CerS1 expression. Restoration of CerS1 upon treatment with FLT3 inhibition resulted in mitophagy-dependent cell death suggesting repression of ceramide synthesis may be an important step in AML pathogenesis. Intriguingly, a mitochondrial targeted ceramide analogue effectively suppressed FLT3 inhibitor resistant AML patient samples in vivo^39^ suggesting the reactivation of mitochondrial ceramide synthesis downstream of FLT3 signalling may be beneficial in overriding FLT3 resistance.

Several groups have observed changes in AC in solid tumours^26,27,80^, with its role in AML recently described. Upregulation of AC mRNA and activity was observed in AML patient samples compared with normal CD34^+^ bone marrow cells^4^. Treatment of AML cell lines with the AC inhibitor LCL-204 was associated with rapid loss of the pro-survival Bcl-2 family protein, Mcl-1 and caspase dependent cell death implicating mitochondrial mediated apoptosis^4^. Although, LCL-204 appeared to display some anti-leukaemic activity in patient-derived xenografts in vivo, the assessment of circulating blasts within the periphery as opposed to bone marrow makes it difficult to draw conclusions from these studies. Overexpression of AC correlated with an increase in Mcl-1 expression and blunted the cytotoxic effects of Bcl-2/Bcl-X_L_ inhibitor, ABT-737. Whilst Tan et al. demonstrate that AC inhibition results in proteasome-mediated degradation of Mcl-1, the exact mechanism of how AC upregulates Mcl-1 was not further explored. Thus, the targeting of Mcl-1 through AC inhibition warrants further investigation in combination with Bcl-2 inhibitor, Venetoclax. Chemoresistant AML cells were also susceptible to cell death induced by AC inhibition which was correlated with the increase in cellular ceramide levels^4^. Although preliminary, these findings serve as a basis for further investigating the potential for AC inhibitors as chemo-resensitisers in the relapse/refractory setting.

The oestrogen receptor antagonist, Tamoxifen has also demonstrated potent inhibition (IC_50_ ~1 μM) of GCS and has demonstrated anti-leukaemic activity. Work by Morad et al. utilising short chain ceramides in combination with Tamoxifen revealed synergistic cell death in AML cell lines as a consequence of mitochondrial metabolic collapse typified by decreases in ATP levels and glycolytic flux^81^. Tamoxifen treatment prevented the accumulation of glucosylceramide species, in agreeance with the proposed role of these molecules as a “sink” for excess cellular ceramide^82^. Besides the demonstrated effects on GCS, Tamoxifen has also been reported to inhibit AC which may contribute to its cytotoxic effects^83^. Nethertheless, the off-target inhibition of GCS by Tamoxifen, presents an interesting therapeutic angle given its approval for hormone-dependent breast cancer, potentially allowing fast track approval as adjuvant therapy in combination with chemotherapy.

Confirmation of SPHK1 as a therapeutic target in AML has been established by several groups using both genetic knockdown of SPHK1 and chemical SPHK1 inhibitors. Chemotherapy resistant AML cell lines exhibited a lack of ceramide generation upon drug treatment suggesting an involvement of SPHK1 as a mediator of drug resistance^16^. Overexpression of SPHK1 in chemo-sensitive AML cell lines imparted resistance to chemotherapeutics confirming SPHK1 as a marker of drug resistance in AML^16,18^. Genetic and chemical inhibition of SPHK1 in AML cell lines and primary patient samples induced cell death^84^ and synergised with chemotherapeutic agents^18^. Interestingly, SPHK1 inhibition-induced synergistic cell death with cytarabine in the refractory leukaemic initiating cell (LIC) population^18^. Although these synergistic effects require further in vivo evaluation, the findings suggest addition of an SPHK1 inhibitor to a standard chemotherapeutic regimen has the potential to greatly enhance clinical responses and reduce relapse rates by targeting the LIC population.

Recently, a link between SPHK1 and Mcl-1 was elucidated by Powell et al. whereby SPHK1 inhibition resulted in Mcl-1 degradation^18^. Loss-of-Mcl-1 coincided with induction of BH3-only proteins, particularly Noxa, a known inducer of Mcl-1 degradation^85^. As Mcl-1 is a marker of resistance to Bcl-2 inhibitor, Venetoclax, this link highlights a new angle to target Mcl-1 and enhance the efficacy of Venetoclax which is exhibiting impressive single-agent activity (ORR 79%) in CLL, a malignancy highly dependent on Bcl-2^65^ but only exhibited modest single-agent activity in AML (ORR 19%)^86^. Follow-up trials with Venetoclax in combination with chemotherapy and hypomethylating agents are currently ongoing with initial reports showing promising results^87,88^. Pre-clinical evidence combining SPHK1 and Bcl-2 inhibition showed synergistic cell death in AML cell lines by targeting both Mcl-1 and Bcl-2^18^. Although, the exact mechanism is currently unknown, the work from both Tan et al.^4^ and Powell et al.^18^, suggests ceramide and S1P regulate Mcl-1 degradation.

A link between ceramide and resistance to Bcl-2 targeting strategies has previously been identified in small cell lung carcinoma which could explain the synergy between the two drugs^89^. Gene correlation analysis for Navitoclax resistance, identified an atypical Bcl-2 protein, Bcl-rambo, as a direct inhibitor of CerS2 and −6^89^. This finding supports in vitro studies demonstrating that the GCS inhibitor, PDMP synergises with Navitoclax suggesting ceramide augments Bcl-2 targeting strategies^7^. The ability of sphingolipid modulating agents such as those directed against AC^4^ or SPHK1^18^ to reduce Mcl-1 expression, as well as the synergy observed with Bcl-2 inhibitors^7,18^, provides considerable impetus to further explore how ceramide modulates Mcl-1 stability.

**Fig. 2:**
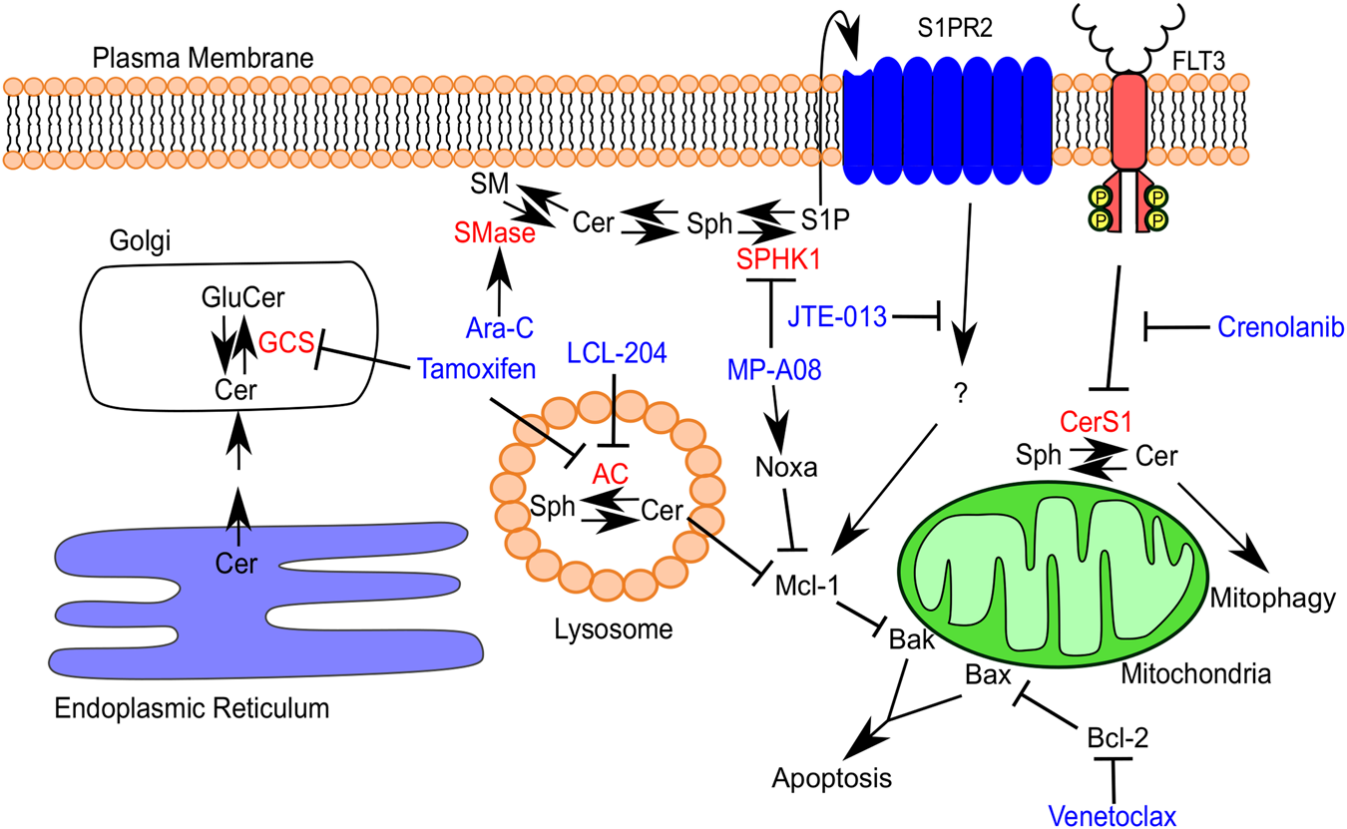
Targeting sphingolipid metabolism in AML. An overview of targeting sphingolipid enzymes (red) in combination with pre-clinical or clinically utilised drugs (blue) in AML.

Targeting of S1P_2_ in AML has shown to induce loss-of-Mcl-1 and synergised with Bcl-2 inhibition recapitulating the observations with SPHK1 inhibition^18^. A link between S1P_2_ and Mcl-1 stability has been largely overlooked in the context of cancer biology. We speculate that a change in Mcl-1 phosphorylation may be responsible for loss-of-Mcl-1 associated with S1P_2_ inhibition highlighting potential involvement of PP2A based on findings observed in CML^51^. Furthermore, this suggests that the loss-of-SPHK1 and its effect on Mcl-1 is two-pronged with reduced S1P/S1P_2_ signalling and the accumulation of ceramide due to the lack of sphingosine processing by SPHK1. Despite the lack of targeted therapies in widespread clinical use, the potentially broad applicability of inhibitors of sphingolipid metabolism in AML may provide significant benefit in a malignancy whose treatment options are reliant on ceramide accumulation for their efficacy (Fig. 2).

### Multiple myeloma

The repertoire of novel therapies to treat multiple myeloma has markedly increased over the past two decades. Of these novel therapies, the proteasome inhibitors (PIs), particularly Bortezomib (Velcade), have resulted in a marked improvement in overall response and median survival rates (from 3 to 6–7 years)^90^. Sustained monoclonal immunoglobulin production by myeloma plasma cells induces endoplasmic reticulum (ER) stress and activates the unfolded protein response (UPR) which aims to reduce global protein translation and correct the misfolded protein-induced stress placed on the ER^91^. To effect this, proteins that cannot be correctly folded are transported to the 26S proteasome for degradation, highlighting the relevance of PIs, which block proteasome activity and commit the myeloma cell to apoptosis due to the accumulation of misfolded proteins in the ER lumen and activation of a terminal UPR^92^.

**Fig. 3:**
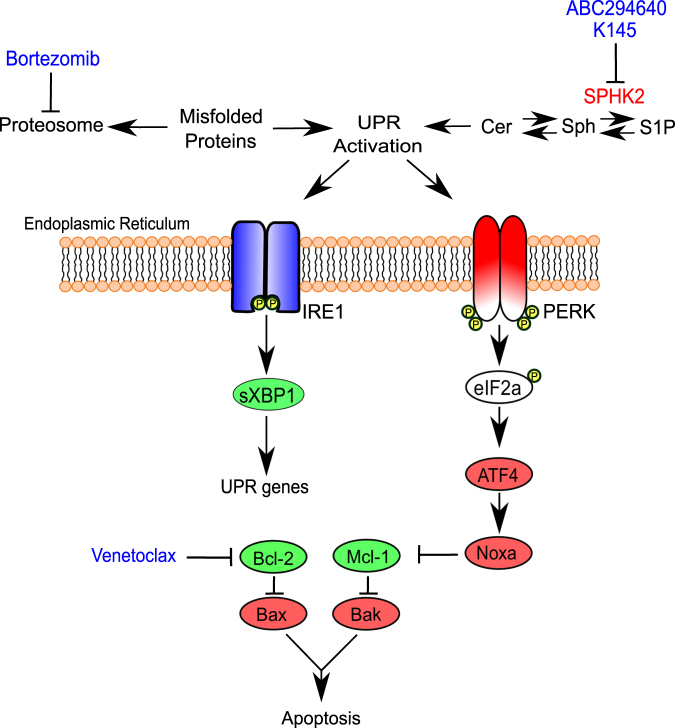
Exploiting sphingolipid synthesis to enhance the efficacy of proteasome inhibitors. Due to the constitutive production of immunoglobulin by malignant plasma cells, their reliance on the unfolded protein response (UPR) to prevent an accumulation of misfolded proteins with the endoplasmic reticulum (ER) for survival renders them susceptible to inducers of ER stress. As the site of de novo sphingolipid synthesis, accumulation of saturated lipids such as ceramide within the ER, induces a lipid dependent UPR, promoting apoptosis.

Unlike other haematological malignancies, SPHK2 appears to be the dominant SPHK isoform in myeloma^5,23^. Both pharmacological inhibition of SPHK2 using ABC294640 or K145, and genetic interference of SPHK2 have shown effects on myeloma cell proliferation and viability^5,23^. In response to ABC294640, a dual dihydroceramide desaturase and SPHK2 inhibitor, loss-of-myeloma cell viability was associated with degradation of c-Myc and Mcl-1, similar to the phenotype observed by Wallington-Beddoe et al. in ALL^22^. The loss-of-Mcl-1, the crucial Bcl-2 family member in myeloma^93^, in response to ABC29460 appears as a consequence of Noxa upregulation^23^, just as it has been shown for Bortezomib^94^, providing evidence for combination therapies. As expected, the loss-of-Mcl-1 observed with ABC294640 treatment sensitised myeloma cells to Venetoclax^95^. Indeed, combination of the SPHK2 inhibitor K145 and Bortezomib has been shown to result in synergistic anti-myeloma effects in vitro and in vivo^5^. These findings are supported by studies showing that changes in lipid saturation in the ER membrane, potentially including the accumulation of sphingolipids, can activate the UPR sensors, IREα and PERK independent of the accumulation of ER luminal misfolded proteins^52,96^. Thus, the basis of synergy likely arises from each drug activating ER stress via distinct mechanisms, culminating in terminal UPR activation and apoptosis (Fig. 3).

## Conclusions

It is well known that sphingolipids are key mediators of cell fate and can rapidly fluctuate in response to drug treatment. The emerging findings from multiple haematological malignancies implicating rewiring of sphingolipid metabolism from different enzymes in the metabolic pathway, such as SPHKs, AC and GCS, highlights multiple therapeutic angles to induce ceramide accumulation. While the majority of drugs targeting these enzymes remain in pre-clinical development, others such as the SPHK2 inhibitor, ABC294640 are under investigation in clinical trials for relapsed/refractory multiple myeloma (NCT02757326). The pre-clinical data suggest that SPHK2 inhibition may augment the efficacy of existing drugs such as Bortezomib and Venetoclax for myeloma^5,23,95^. The success of ABC294640 may also accelerate other SPHK inhibitors into clinical trials. Furthermore, the findings that clinically used small molecule inhibitors of FLT3^39^, BCR-ABL^17^ and Bcl-2^67^ increase ceramide levels warrant further investigation. In particular, the combining of two separate drugs such as the FLT3 inhibitor, Quizartinib and the Bcl-2 inhibitor Venetoclax which have demonstrated synergistic cell death in AML, could be partially attributed to the increases in ceramide levels that are produced by each drug^39,67,97^. Thus this could provide an opportunity for targeted combinational therapies under the premise of synergistic increases in ceramide levels. Finally, repurposing approved drugs such as Tamoxifen, which has activity against AC, GCS and SPHK1 could prove beneficial, particularly in the relapsed/refractory setting where each of these enzymes can potentially confer drug resistance. Given many of the drugs used in the clinic modulate ceramide levels, herein provides an opportunity to partner existing agents with rationally chosen sphingolipid inhibitors to collectively induce lethal levels of ceramide that target tumour cells and enhance patient survival.
