# Author Correction: Targeting sphingolipid metabolism as an approach for combination therapies in haematological malignancies

**DOI:** 10.1038/s41420-020-00380-1

**Published:** 2021-06-18

**Authors:** Alexander C. Lewis, Craig T. Wallington-Beddoe, Jason A. Powell, Stuart M. Pitson

**Affiliations:** 1 Centre for Cancer Biology, University of South Australia and SA Pathology, UniSA CRI Building, North Terrace, Adelaide, SA 5001 Australia; 2 Adelaide Medical School, University of Adelaide, Adelaide, SA 5000 Australia; 3 Flinders Medical Centre, Bedford Park, SA 5042 Australia; 4 College of Medicine and Public Health, Flinders University, Bedford Park, SA 5042 Australia

Correction to: *Cell Death Discovery*

10.1038/s41420-018-0075-0 published online 28 June 2018

Since online publication of this article, the authors noticed errors in the labelling of some of the arrows in Fig. 1, which shows a schematic of the sphingolipid pathway. The corrected image is provided below. The authors apologise for any inconvenience caused.

**Figure 1:**
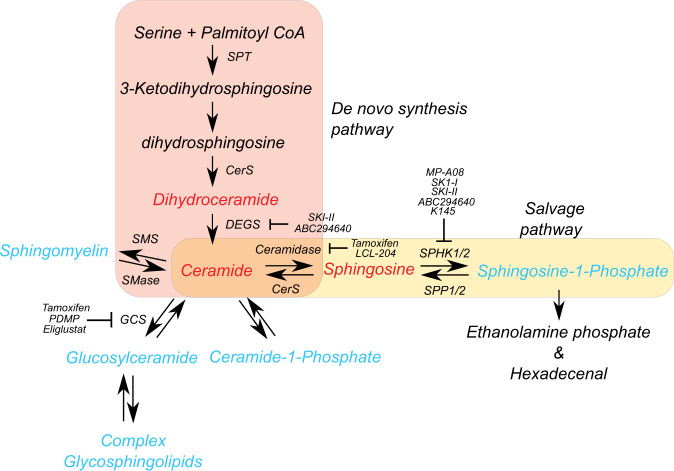
Fig. 1.

